# On Strictures of the Rectum

**Published:** 1845-03

**Authors:** Thomas D. Mütter

**Affiliations:** Professor of Surgery in Jefferson Medical College, Philadelphia


*On Strictures of the Rectum.* By THOMAS D. MÜTTER, M. D., Professor of Surgery in Jefferson Medical College, Philadephia.

Continued from page 89.

MALIGNANT STRICTURE.

Under this head we include all the morbid changes to which the intestine is liable, which present the usual characteristics of malignant disease in other parts of the body. According to the observations of Bushe and others, it would appear that the complaint manifests itself under one of three garbs, and my own observations tend to prove the correctness of their views. It is either *schirrous*, resembling in feel and consistence cartilage; *lardaceous* or cheesy; or *encephaloid.* The most common variety is unquestionably the simple schirrous, but I have seen this combined with the lardaceous deposites; and again have met with a case, schirrous in its origin, gradually converted by a higher degree of organization into the encephaloid. Mayo states that he has seen the disease but under two forms—one, in which "the mucous membrane is abraded, the muscular coat hard, firm, gristly, and the canal of the bowel narrowed. The disease usually occupies several inches of the intestine, and terminates by an uneven ulcerated edge." Another, "fungoid in structure, originating as a hard tumour, gradually increasing, becoming softer, and finally ulcerating."

When the disease is schirrous, it may develope itself in the form of *hard tubercles*, which are deposited either in the mucous or cellular coat of the intestine, and then gradually involve the others, or as a *general infiltration*, as it were, of the muscular coat first, which causes its condensation and thickening. From either, there is ultimately established a firm, rigid, and almost cartilaginous tissue across the rectum, in the centre of which there is usually detected an irregular orifice, with margins softer and more yielding than the other portions of the obstructions.

In the *lardaceous* deposite, one or more, and sometimes all of the coats of the rectum, are converted into a substance like soft cheese, white in colour, and readily broken down with the finger. This deposite, by thickening the walls of the intestine, cause them to encroach upon its cavity, and thus occasion a stricture.

The *fungoid* or *encephaloid* variety may originate from a schirrous or lardaceous deposite, or it may be a primary formation. It usually presents the shape of a circumscribed tumour, softish, easily broken down, and resembling encephaloid matter in other parts of the body.

Any portion of the intestine may be the seat of these deposites, but as yet it is not positively ascertained which part is most liable. Some contend that the lower third is usually the point of attack, and this corresponds with my own observations; but I have seen the middle, and upper third primarily involved, while the lower was entirely exempt for several months.

The extent of the stricture also varies in different cases—usually but two or three inches of the intestine are affected, but sometimes we find its entire length included.

According to the observations of the celebrated Dessault, malignant disease of the rectum is by no means uncommon in persons advanced in life, but other authorities appear to think that middle age is the period at which it usually makes its appearance. Occasionally, though rarely, it is met with in young persons. Mayo, Bushe, and others report cases of this kind.

With the exception of Calvert and a few others, all unite in declaring the disease to be more common in the female than the male; and various explanations of the fact have been attempted. The varying condition of the womb during utero-gestation; its periodical conjestion in the unimpregnated state, which extends to the rectum; the want of support to which the rectum is subjected by the straightness of the sacrum and the general expansion of the pelvic bones in the female; and the irregular habits of many women in reference to the calls of nature, have all been considered as exerting a predisposing influence. Certain it is, that the disease usually makes its appearance about the cessation of the catamenia, which indicates some connection between the disappearance of this secretion, and the developement of the cancer.

With respect to the proximate cause of this complaint, there exists as yet nothing definite. That local injury may occasion its manifestation, no one questions, but it appears to me that in the majority of such cases, there must exist some tendency, either hereditary or acquired, to cancerous affections, and that the local cause merely hastened the development of the disease. By some, especially Lisfranc, the disease is considered as strictly local in its character.

Although malignant complaints of the rectum are usually attended, even from the first, with pain and distress of various kinds, the progress of the disease is by no means the same in every case—sometimes it grows with great rapidity, but for the most part its progress is slow, patients living for months and years, and ultimately sinking from exhaustion alone.

The symptoms indicative of this affection are very much the same in the different forms of malignant disease, being modified of course by the location, extent, and complication of the complaint. The first thing complained of by nine out of ten patients, is costiveness, accompanied in most cases by a severe burning or lancinating pain, or a dull aching sensation in the rectum, which shoots through the pelvis, or extends up and down the loins and thighs. The pain in the thighs is often agonizing, and sometimes alternates with a *numbness*, or a sensation similar to that we experience when a part is "asleep." There is also a sense of weight in the back and loins, aching above the pubes, and often spasm of the anus; usually, though not invariably, these symptoms are all aggravated by exercise, and relieved by rest in the horizontal posture. There is also in some cases troublesome anorexia, abdominal pain, flatulence, and irritation of the bladder. As in simple strictures, the bowels here are in an exceedingly irregular condition, costiveness alternating with diarrhœa. The fæces are generally liquid, or broken into fragments, but sometimes they are natural in appearance and from the same cause that operates in simple stricture. The evacuations, when the costiveness yields, are always frequent and scanty, and usually mixed with mucus, blood, or pus,—and in the advanced stages with shreds of flesh or sloughs. When the disease extends to other organs, we have, of course, a new train of phenomena developed, which are for the most part sufficiently characteristic and to be recognised at once. Thus the irritations of the bladder, uterus, or bowels, when they occur, are readily distinguished. As the case advances, all the symptoms enumerated are increased in intensity,—but there is often added profuse hemorrhage from ulceration of the bowels, immense secretions of pus, the development of abscesses communicating with adjacent viscera, fistulæ, and sometimes dreadful destruction of the soft parts in consequence of ulceration and sloughing. I have known patients, however, destroyed by this disease, in whom no ulceration or sloughing occurred. The general health of the poor victim necessarily yields to the terrible influences of the local complaint—and along with the dyspeptic symptoms, the usual accompaniments of the disease, his complexion becomes sallow or leaden coloured, his limbs œdematous, the glands in the vicinity of the rectum enlarged, until at last, death, now a most welcome visitor, closes an existence the most wretched that can possibly be imagined.

It is often a matter of great difficulty to determine, in the commencement of the case, between a simple stricture and incipient cancer. Usually in the latter, there is the sharp, lancinating pain, even in the first stage; and when examined with the finger, it appears more firm and unyielding than the simple variety, at least such is the fact when *schirrus* exists. When the *lardaceous* or *fungoid* deposites are present the diagnosis is more difficult, because the obstruction is *soft* and *yielding*; but usually this softness is much more decided than when the case is one of simple stricture; and there is usually a much greater disturbance of the general health—the patient presenting the peculiar aspect of one suffering from malignant disease. In the advanced stages, the severe pain, the profuse discharge, the rough surface, either hard or softish, the early contamination of adjacent parts, and the great constitutional disturbance, will be sufficient for us to form a correct diagnosis as soon as an examination of the case is made.

I once knew an ignorant physician mistake the disease for a common tumour or polypus of the rectum; but no one at all familiar with the two affections, or who has ever read a description of their peculiarities, could commit so gross an error. In the interesting cases of Mr. Crosse of Norwich, and Mayo, in which the fungoid disease manifested itself in the shape of a single tumour, with a well defined and circumscribed base, the malignant tendency was at once recognised, and although the tumours were removed, an unfavourable prognosis was given, and the disease eventually returned.

There is also another affection of the rectum and anus, first described by Cayol and Bayle, with which cancer is liable to be confounded. It resembles in all its attributes the "*Elephantiasis of the Arabs*," and is distinguished from schirrus by its development being accompanied with distinct febrile attacks, during which there is severe pain in the part, and the swelling increases; by the absence of much constitutional disturbance, until the disease has acquired considerable volume; and by the fact that the *hardness* of the tumour is much greater externally than within the rectum. Dissection, too, readily reveals the difference between the two complaints.

Assuredly there is no complaint in which the prognosis is more unfavorable, for there is scarcely a well authenticated case of recovery from its attack. The few examples of alleged cures from excision of the diseased portion, do not deprive the above assertion of its weight; for although the patients were benefitted *for a time*, there was in nearly every instance a return of the disease. The measure is therefore to be considered, when justifiable at all, a palliative only.

The fatal result is brought about in one of three ways. The disease either gradually consumes the patient by long continued suffering, or it destroys him by producing complete obstruction of the bowels, or it occasions fatal peritonitis. Usually the schirrous and lardaceous varieties terminate in the first manner, while the fungoid is most apt to obliterate the passage—either may occasion peritonitis.

The post mortem appearances in one of these cases, are dependant of course upon the form of the complaint. Thus we may have schirrous, lardaceous or fungoid masses, or all combined, and confined to the coats of the rectum, or extending to the neighbouring organs. Usually the rectum is bound by close adhesions to the bladder or vagina, and it is most important to keep this fact in mind when we have to decide upon the propriety of an operation. The extent of the disease, as I have already said, varies in different cases—sometimes only an inch or two of the rectum being involved, while at others the whole intestine seems to be attacked—and again the deposite may take place in distinct patches or spots.

*Treatment.*—The surgeon should ever bear in mind in the treatment of schirrous rectum, that his remedies in the vast majority of cases are but palliatives. Yet he should equally recollect that these palliatives are often of immense utility, prolonging, as they frequently do, the life of the patient, and in all cases materially assuaging his sufferings. There is in fact no form of disease in which the benefits to be derived from a judicious course of management stand forth more conspicuously. However irksome, then, may be the attendance upon such a case, the surgeon is in duty bound to stand by his patient to the last.

One of the most important indications to be observed inthe treatment of the case, refers to the condition of the bowels. These should be kept in an easy condition by laxative diet, if possible; but when this fails, the mildest cathartics, as oil, ccnfection of senna, lac. sulphuris, and supertartrate of potassæ, should be employed; and their operation aided if necessary by enemata of warm milk and water, linseed oil and lime water, warm castor oil, or flaxseed mucilage. Active purgation, however, from the fact that it always increases the irritation of the parts, must be carefully avoided; and a very good rule is to administer a small dose of laudanum or morphia, as soon as the bowels have been once or twice relieved. Too much confidence cannot be placed in the castor oil injection, for not only does it act as an evacuant, but often it appears to operate as an anodyne, soothing the parts and removing the pain. I have often observed this in other cases of cancer, and I constantly order it as a local application in ulcers of this type in other parts of the body.

The severe pain with which most patients are afflicted, must of course be relieved by anodynes taken by the stomach and employed in enemata. It appears, probably, in consequence of the functions of the rectum being more or less impaired by the disease, that enemata do not answer as well as when the medicine is taken in the usual manner; but we often find it necessary to resort to both forms of administration. Much reliance was at one time placed on the use of cicuta and hyoscyamus, but I believe we shall gain more from opium and morphia than any thing else. When, however, these articles appear to lose their effect, it is well to alternate them with other anodynes. Great benefit, too, results from the occasional use of the warm hip bath; but when the patient is much debilitated it cannot be employed with either advantage or safety. Much confidence has likewise been placed in the repeated application of leeches to the anus, but so far as my own experience goes, I have rarely seen any benefit derived from their use. On the contrary, they weaken the patient, render his nervous system more excitable, and thus increase his sufferings. Consequently, I never employ them unless there exists decided inflammation of the parts. Frequently much good results from small doses of balsam copaibæ, and diluent drinks, and when there is flatulency or acidity of stomach, the different alkalies must be employed. The diet of the patient should be mild and nutritious, and as a general rule he must be kept in the horizontal position as much as possible. When, however, we find that he is losing strength from the want of fresh air, it is necessary to send him out in a carriage whenever the weather will permit. Many attempts have been made to relieve this disease by different alterative remedies, so called, as mercury, iodine, arsenic, sarsaparilla, &c., but without much success. I think, however, that I have derived decided advantage from iodine in combination with sarsaparilla, and I generally employ the following formula.

℞ Potass. Iodid., ʒij.
Syrup. Sarsaparill. Comp., ℥xvi.
M. Sig.—A tea-spoonful to be taken three times a day.

I have also, at least so it appears to me, gained a good deal in cases attended with much inflammation, by the use of digitalis in the usual dose. In the different forms of schirrous inflammation, I am sure that nothing is more to be relied on, as I have seen it in cancer of the breast, for example, change the character of the ulcer in the course of a few days, and ultimately cause its cicatrization. Its beneficial effects were most decided in a case sent me from Easton, and which is now under the judicious and skilful management of Dr. Stout of that place. It probably acts by diminishing the quantity of blood determined to the seat of disease. Great differences of opinion are entertained relative to the employment of *bougies* in the treatment of cancer of the rectum; some declaring that, so far from doing good, they are either nugatory or positively hurtful, while others employ them in every case. If those opposed to their use will only tell us how we are to prevent the closure of the passage without them, at least in many cases, I for one would be most willing to dispense with the measure; but until that is done, I am forced to declare that the *cautious* and *occasional* introduction of the bougie, is not only most useful, but often absolutely essential. The air bougie, or the ordinary rectum bougie, is the instrument to be employed, and must be introduced just as in cases of simple stricture; but I prefer, whenever it can be employed, the forefinger. With the finger we can feel our way cautiously, and there is no risk of rupturing the intestine or injuring it in any way,—and being somewhat yielding, it can be introduced with less suffering than a bougie of the same size. It must be introduced two or three times a week—that is, if the patient can bear it thus often, and merely passed through the stricture.

Occasionally we meet with strictures so close, and with a sensibility so acute, that neither the finger nor bougie can be borne. In all such Mayo has proposed to divide the obstruction with a probe-pointed bistoury. I have never myself been under the necessity of resorting to the measure, and cannot therefore speak from experience as to its merits, but in Mayo's case the relief was so decided, and the risk so trifling, that I am disposed to advise its employment under similar circumstances.

As the disease advances, it becomes necessary to support the strength of the patient by tonics, a good diet, &c., and also to increase the dose of anodyne, notwithstanding the injury inflicted by it upon the digestive apparatus and nervous system. Without anodynes, life would prove a burthen too onerous to be borne.

Occasionally we find indications of inflammation of the rectum both in the early and more advanced stages of the complaint, and then the application of a few leeches to the anus is often exceedingly useful; but this measure is not to be employed unless the necessity is absolute, for few patients can bear the loss of blood. Of course, the antiphlogistic treatment must be observed until the inflammation has disappeared. Often it occurs, too, that fæcal retention takes place, and then we must resort to the introduction of a flexible tube, (through the stricture,) by means of which, and a syringe, we soften the fæces, and afterwards wash them out of the canal.

EXCISION OF THE RECTUM.

From the acknowledged incurability of cancer of the rectum by the usual methods of treatment, it has been proposed to excise the diseased tissue entirely, just as we do in other cases of cancer. It appears that this operation was mentioned by Morgagni, as the unsuccessful project of some surgeon of his day; but Beclard, in 1822, was the first surgeon of authority who advocated its performance. Before this, however, Faget, in

1739, performed the operation with success, removing one inch and a half of the intestine. Notwithstanding the advice of Beclard, who, by the by, never performed the operation himself, no one seemed disposed to undertake it until 1826, when Lisfranc took up the subject, and by his reputed successes, gave a decided eclat to the practice. Since his publication, the profession has been very much divided as to the propriety of the measure, but the weight of authority seems to be against it, in consequence of the liability to relapse, and the hazards of the operation itself. Nearly all unite, however, in this, that the operation is only justifiable *when the limits of the disease can be reached by the finger; when no adhesions exist between the rectum and surrounding parts, which would prevent the bowel from descending when the attempt to draw it down is made; when the case is recent; and finally, when the general health of the patient is such as to warrant the performance of so severe a measure.* Under no other circumstances should it be undertaken; nor even here, without first informing the patient of the little probability of success,

The following is the best mode of proceeding when it is deemed proper to operate.

"The patient being placed in the position usually chosen for lithotomy, two semilunar incisions are made about an inch from the anus, on each side of it. These cuts, which are to extend through the skin and cellular tissue, are to unite behind and in front of the rectum. The end of the bowel is then to be separated by dissection from all the surrounding parts. The fore finger, half bent, is then passed into it, for the purpose of drawing it downwards, and, by this means, the mucous coat, which may be the part alone or principally diseased, may be made to descend a good way; and a considerable portion of it may be easily removed with scissors curved sideways, or with a bistoury. Even if the cancerous affection should implicate the whole thickness of the parietes of the rectum, the advocates of the operation assert, that the rectum may be turned inside out, and the whole of the disease brought into view, provided it does not extend more than an inch above the anus.

"When all the coats of the bowel and some of the adjoining cellular tissue are involved, the surgeon, after making the semilunar incisions, and detaching the end of the bowel at the whole of its circumference, is to pass his fore finger into the bowel, in order to serve as a guide for a pair of strong straight scissors, with which the gut is to be divided parallel to its axis, through its entire thickness, as far as the limits of the disease. This incision is to be inclined towards the posterior wall of the bowel, so as to be further from the vessels and the peritoneum. The latter cut enables the surgeon to unfold the gut, and expose the disease through its whole extent; but if it should be concealed by the hemorrhage, a sponge full of cold water must be held for a few moments on the wound, for the purpose of stopping the flow of blood; and the lower portion of the rectum is to be kept downwards with hooks.

"In operating on a female, the assistant's finger introduced into the vagina will be of service. When the patient is a male, it is prudent to introduce a catheter into the bladder, which should be intrusted to an assistant."—(S. Cooper.)

The two dangers of the operation are *hemorrhage* and *peritoneal inflammation*, both of which, should they occur, are to be treated on general principles. The dressings of the wound should be a light pledget of oiled lint, which is daily renewed; and after all danger of inflammation has disappeared, the advice of Lisfranc, "to cause the patient to wear a thick roll of lint, which distends the canal," may be adopted, and continued for several weeks. Although incontinence of the fæces may occur after this operation, it is yet surprising how much, in many cases, is accomplished by nature towards the establishment of a substitute for the natural canal. A kind of sphincter is formed out of the circular muscular fibres of the rectum, and some of those of the levator ani, which retains the fæces, and the canal from which we have removed the intestine is soon lined by a membrane very similar to mucous tissue. In the case of Mayo and some others, the patients lived for two or three years, and during the whole of this period were able to retain their fæces, and suffered little or no inconvenience from the operation.

Notwithstanding these favourable results, it should be borne in mind that the operation, in the vast majority of cases, is altogether unjustifiable, and should never be undertaken unless the favourable circumstances already referred to are present.

### THE ESTABLISHMENT OF AN ARTIFICIAL ANUS.

When all the remedies enumerated fail to relieve the patient, and the obstruction is such as to produce dangerous retention of fæces, surgery, fertile in resources, still offers another mode by which, in some cases, life may be prolonged, viz.: the "establishment of an artificial anus."

As this operation is comparatively modern, it may not be uninteresting to glance at its history. It appears that some of the ancients, especially Praxagoras, proposed a measure somewhat similar to the one now in use; but unquestionably Littré, in 1710, was the first of the more modern authorities to suggest the establishment of an artificial anus in cases of insurmountable obstruction of the rectum. According to the statement of the able writer of an excellent article upon this subject in the October number of the British and Foreign Review, there has been a most singular misinterpretation of the words of Littré, who merely proposes "opening the cavity of the abdomen, dividing the rectum, and then connecting the upper portion of the intestine with the external wound!" He does not state the position in which the wound should be made, although he is reported to have advised the *sigmoid flexure of the colon*, to be reached through the iliac region, as the most favourable point. But notwithstanding the recommendation of such a measure, it appears that Littré never performed the operation, which was first executed by Pillore, of Rouen, in 1776, who made his incisions so as to reach the *cœcum*. The patient who had cancer of the rectum survived only twenty-eight days. Fine, of Geneva, was the next, in 1797, to perform a somewhat similar operation for cancer of the rectum, but he opened the *transverse colon* from the *umbilical region*. Since this operation, the method of Littré, slightly modified, has been put into practice by Freer, in 1818—Pring, in 1820—Martland, in 1824—Velpeau, in 1839—Amussat, in 1840—and Thierry also in 1840. Although these operations serve to fulfil the indication of relieving the fæcal retention, they are defective in one most important point; they *all involve the peritoneum*, and hence the danger from inflammation of this membrane is always very great. Indeed, with the exception of Martland's case, whose patient was alive seventeen years after the operation, and Pring's, in which the patient survived six months or more, and Fines', where death occurred after the lapse of three months and a half, the operation has proved fatal in a few days, at least where it has been performed for cancer of the rectum. When performed for imperforate anus, it appears to have been a little more successful, as is shown by the cases of Duret, Desgranges, Serrand, Miriel, and Klewig, but here, too, the mortality has been very great.

As early as 1796, Callisen saw the objection to the plan of Littré, and proposed to open the *descending colon behind*, from the *left lumbar region*, where it is not covered by peritoneum. It does not appear that he ever performed the operation on the living subject, and it remained for Ashmead, of Philadelphia, and Amussat, of Paris, to bring the measure fairly before the profession. Although M. Amussat has generally received the credit of having first succeeded by this process, it is yet certain that Dr. Ashmead performed his operation on the *15th of March*, 1838, and that Amussat reports his as having taken place on the *2d of June*, 1838. Both are modifications of that of Callisen.

Dr. Ashmead has operated, I believe, in *two* cases, but the patients died in a few days. Amussat has had *six*, and of this number one lived *five months*, one was alive *two years* and *three months* after the operation; another *ten months after;* another *seventy-five days* after; another *a month* after; while the sixth died on the tenth day. Baudens has operated *once*, and the patient died on the fifth day; Clements *once*, and the patient recovered; Teale *once*, and death occurred on the seventh day; Jukes *once*, and death ensued on the tenth day. It must be confessed, that such a resumé is anything but satisfactory.

*Operation as performed by Amussat.*—The operation as performed by Amussat, is executed in the following manner:

"A transverse incision is to be made two fingers breadth above and parallel to the crista ilii of the *left* side, or rather in the space which is bounded by the false ribs above, and by the crista ilii below; the incision should commence at the external margin of the erector spinæ, and extend outwards for about four inches. The spinous processes of the lumbar vertebræ, the crista of the ilii, and the last false rib, are the principal guides. The superior margin of the crista ilii is, however, the safest of these; and the transverse incision may be said to correspond to the *middle third* of this part of the ilium. After having divided the skin and all the more superficial tissues, the deeper layers are next to be incised as they present themselves; if necessary, the external border of the quadratus lumborum may also be cut across. The dissection is then very carefully to be carried through the layers of cellular tissue which lie immediately upon the intestine, and the colon sought for; this will, in general, readily present itself, and may at once be recognised by its colour and distended appearance. The operation may then be completed by passing a tenaculum, or needle armed with a strong waxed thread, into the most projecting part of the intestine, and by this means drawing it to the surface of the wound, in order to prevent it shrinking or sinking back when opened; it is now to be punctured with a large bistoury or trochar, and its contents having been evacuated, the sides of the opening in the intestine are to be fixed to those of the incision in the skin by four or five points of suture, so as to prevent the contents of the bowel being effused into the cellular tissue of the wound. It is highly important to draw the colon well forward before opening it, in order to prevent its contents escaping into the wound, where they would set up considerable irritation, and prevent or retard the union of the parts. There are but few vessels and nerves cut, as they for the most part run parallel to the line of incision. When the operation is practised on the dead body, it will be found on dissection that the following are the parts cut through.

After the *skin* and *cellular tissue*, the *latissimus dorsi* will be seen divided towards the posterior third of the incision, and the *obliqus externus* in the *anterior* two-thirds of it, the *obliqus internus* and the *transversalis*, sometimes the *quadratus lumborum*, the *cellulo-adipose* tissue which immediately covers the intestine, and finally, the *colon* itself."

*Operation of Ashmead.*—The following extract explains the method of operating resorted to by Dr. Ashmead, and it must be obvious to all familiar with the subject, that it is almost identical with that proposed by Callisen. It is due, however, to Dr. Ashmead to state, that at the time his operation was performed he had never seen or heard of the method of the surgeon of Copenhagen.

"An incision was made through the skin, extending from the last rib to the crest of the ilium, immediately anterior to, and parallel with the sacro-spinal mass of muscles—the subcutaneous cellular tissue and superficial fascia were next divided with the aid of a grooved director. Three or four large nerves, and two small arterial and venous trunks now presented themselves, crossing the wound within its inferior half; the arteries and two of the nerves had to be divided, but no hemorrhage ensued, in consequence of the sudden retraction of the distended vessels. A layer of cellular tissue being now divided, brought into view a thin fibrous aponeurosis, which is the internal layer of the transversalis fascia, given off from its posterior edge to the internal surface of the quadratus lumborum, and inserted at the roots of the transverse processes of the vertebræ. This tissue is of uniform appearance and thickness, lies next to the bowel, is attached to it by cellular tissue, and might readily be mistaken for peritoneum—behind it laid the bowel enormously distended, but not visible, owing to the intervening fascia. At the inferior angle of the wound the edge of the quadratus lumborum was divided, in order to prevent it from forming a pouch with the punctured bowel.

"Without dividing this aponeurosis, a large temporary ligature, in a strong curved needle, was passed through it, and the presenting portion of bowel, about an inch above the crest of the ilium, entering from behind, and brought out three-quarters of an inch anterior to its point of entrance. A second ligature was immediately placed about one inch and a half above the first. The ends of these ligatures were now secured to the edges of the wound, on both sides, in order to fix the bowel firmly, and to prevent infiltration in the interstices of divided parts or within the fascia. A small perpendicular opening was now made, by a bistoury, into the bowel, between the two ligatures, and enlarged by the aid of the director and finger to the length of one inch. Instantly a volume of gas and a small quantity of thin feculent matter escaped. All vomiting, spasm, and acute pain immediately ceased upon the opening being made. A piece of large flexible catheter was introduced about two inches up the colon, and secured by adhesive strips—(a measure which subsequent experience proved to be useless.) The application of simple dressings around the wound, and a soft sponge to its edge to receive the discharge, completed the operation.

"On the third day the catheter was removed, and the ligatures were withdrawn at the end of a week. As the wound contracted, its *posterior* edge was drawn towards the spine, while the surface next the abdomen, owing to the elasticity of the surrounding parts, was drawn along with it, so as partially to overhang the opening into the bowel, thus forming a sort of imperfect valve, and giving the opening an oblique direction—without at the same time interfering with the evacuations. The mucous membrane of the bowel was evoluted, forming an elevated ring around the wound, which was extremely sensitive to the touch.

"The operation was followed by considerable prostration for three or four days, owing to the sudden removal of distension, but all vomiting and pain ceased, the patient was able to take nourishment, and slept comfortably, with but little aid from anodynes. The change was so striking and gratifying that her friends now entertained hopes of a recovery; but the abdomen continued larger than natural, though it was soft and flabby—at the end of ten or twelve days a diarrhœa set in, with loss of appetite and depressed spirits, and the patient sank on the sixteenth day after the operation.

"A *post-mortem* examination, hastily made, revealed universal adhesions of the peritoneal surfaces of the bowels, with false membrane in some places one-eighth of an inch thick, limpid serum in the cavity of the abdomen—coats of the intestines so much thickened as to have lost their elasticity—no pus about the wound—the incision at least half an inch from the peritoneum, on either side of the bowels. Schirrus of the rectum, four inches in extent, extending to the vagina, arteries, ovaries and bladder.

"Dr. Ashmead believes that his patient might have recovered, had not organic disease existed to so great an extent before the operation."

All surgeons acknowledge the hazards of either operation, but there is a difference of opinion in relation to the *comparative danger* of *each.*

The advantages of the process of Callisen are,

1*st.* The peritoneum is not opened, and hence there is less risk of peritonitis.

2*d.* As only *one side* of the colon can be drawn forwards, and not a *knuckle* of it, as would be the case if the *small* intestines were operated upon, it is evident that the spur-like process, which exists in all such cases, would present to a very slight degree; and consequently, if the *artificial* anus become useless by the restoration of the natural passage for the fæces, it could be readily closed without any fear of the ridge or spur, which is present in common artificial anus, occasioning any obstruction to the descent of the contents of the intestinal tube.

3*d.* If the peritoneum is accidentally wounded in our attempts to reach the colon, the wound will be at the most dependent part of the abdominal cavity; and hence the danger from effusion of fæces, pus, &c., would be much less than when the opening is made in front.

4*th.* It is said that there is less risk of prolapse of the intestine.

All these alleged advantages are denied by Vidal and others, who still prefer the operation of Littré; but the weight of authority is at the present time decidedly in favour of the method of Callisen, or its modifications by Ashmead and Amussat.

The orifice which remains after the union of the parts is situated at the bottom of a *deep pit*, is lined by mucous membrane, and must be kept covered by a sort of truss or bandage, which should be removed whenever the fæces are discharged. If this orifice contracts, it should be dilated with sponge tent, and when the mucous membrane prolapses it must be returned, and carefully supported by the bandage.

Many surgeons contend that the hazards of these operations are such, and the inconvenience attendant upon an artificial anus so great, that the measure is scarcely justifiable. But it is our duty in these terrible cases to offer the patient or his friends every resource of our art; and it is for them to determine whether or not life, under all the circumstances, is desirable. Many persons have survived for several months or even years, and have lived to settle their affairs, or bring up their children even, who, without the performance of the operation, must have died in a few days. I cannot, therefore, hesitate to advise a recourse to the measure, when every thing else has failed, and death inevitable in a few days without it.

